# Proper Care of the Eyesight

**Published:** 1891-07

**Authors:** Lewis S. Dixon


### PROPER CARE OF THE EYESIGHT.[1]

BY LEWIS S. DIXON, M.D.

MR. PRESIDENT AND GENTLEMEN,—I have been honored by an invitation to give a few suggestions as to the proper care of the eyes and their preservation from unintentional abuse. At a time when one's attention is called in so many directions by the ardent advocates in many specialties, and the advice given is too abundant for any attempt to follow it all, it may seem superfluous to ask your thoughtful interest to this special subject.

But there are peculiar reasons why this topic should claim a little thought. First, because in your profession accurate vision and free use of it are matters of such direct importance in enabling you to apply your knowledge and obtain the good results and finish of workmanship that you desire. Secondly, because abnormal or imperfect vision adds such a decided and complicated burden to a work, of necessity, fatiguing and laborious. Thirdly, because the conditions of imperfect or faulty vision can be so positively detected and proved and so thoroughly corrected and relieved; for the advances made in certain branches of ophthalmology have carried us out of the region of probability and theory into that of almost mathematical certainty.

I am aware that, in speaking to those who have already in their studies paid more or less attention to the anatomy and physiology of the eye, I may need your kind indulgence for repeating much that is already familiar to many, but there are one or two fundamental points which it is of great importance to understand and realize, which experience shows are, as yet, but dimly recognized

---

[1] Read before the American Academy of Dental Science, Boston, March 4, 1891.

even by physicians, and yet they underlie and account for a large portion of the troubles commonly affecting the use of the eyes.

As a part of the body, and one of the organs of sense, many and complicated diseases may affect its various parts. Most of these are evident at once by external or easily recognizable symptoms; others, less common, affecting the deeper parts, not so evident, yet still almost always accompanied by such impairment of vision or definite indication of trouble as to call for attention. All these fall naturally under the care of the physician, and their diagnosis and treatment are to a great extent under the same limitations and uncertainties as hold elsewhere in the body. Such difficulties are of course not included in this paper. There are, however, a very large number of people who, having very good vision, or what they suppose is good vision, and no apparent disease, have more or less complaint to make, saying that their eyes are weak, incapable of comfortable or continued use without pain or discomfort afterwards. This leads them to feel it necessary to limit the use of their eyes, for they realize that in some way their eyesight is not quite as they wish it to be; and yet, as no apparent cause is discoverable, and vision good, they rest content with weakness as a sufficient explanation. Also, there is another large class, who, with excellent sight and no symptom calling attention to the eyes, suffer much from headaches, sick headaches, neuralgia, undue fatigue, and nervous exhaustion,—who strive in vain for any real relief through medicines and treatment, and are totally unaware that the whole cause lies unsuspected in the eyes.

It is, therefore, very necessary that, in order to take proper care of the eyes, one should know the meaning of the various signs that may be noticed, and be able to recognize the symptoms of overwork or strain and understand how it comes about.

For almost every one who has fair sight, it is very difficult to realize that seeing necessarily involves any muscular effort, unless it may be when looking at objects very near. Simply to open the lids seems all that is needed. This is practically true of really normal eyes, but, unfortunately, this class is much smaller than is supposed, and many who seem warranted in boasting of their fine vision are greatly surprised to find themselves not included. The important point to remember is, that sight involves two processes, entirely distinct from one another: first, the focussing the light from the objects looked at, so as to produce an actual picture on the retina; secondly, the perception or feeling of this picture by the retina or nerve of sight. The first is entirely an optical process, controlled and limited by the laws of optics. The second is entirely a physiological process of the optic nerve and depends on its healthy action, which, fortunately, is the usual condition. The poor optic nerve has been greatly maligned, and all the impairments of vision whose causes were not evident or otherwise explained have been laid to its charge, but, unless on account of occasional disease or exposure to excessive light, it always acts well, and practically without fatigue.

Simply as an optical instrument, however, the eye may have numerous and complicated defects, which are entirely optical and fall under fixed laws capable of demonstration and proof. And so, unexpectedly perhaps, these optical errors must be understood before the meaning of the various symptoms and complaints in regard to the eyes can be made clear. The optical function of a normal eye is exactly that of a photographer's camera,—to refract or bend the light received from objects to a focus and produce a sharply-defined picture—in the camera, on the prepared plate, so that it may be taken; in the eye, on the retina, so that it may be felt or perceived.

It is well known that the photographer has to use great care in adjusting the focus of his camera for each picture he wishes to take, the slightest variation of light or distance of the object needing corresponding changes in the adjustment. This is due to the fact that the rays of light proceed from each point like the rays from a candle-flame, diverging in all directions, and that the nearer the lens of the photographer or of the eye is to the luminous object, the larger the cone or the more divergent the rays which will pass through it, and that as the object is removed, the cone becomes smaller and smaller, till at last the rays become practically parallel.

The normal eye is so built, or formed, that its lens, immediately back of the pupil, is just strong enough—*i.e.*, has the proper bending power—to exactly focus parallel light or that from distant objects upon the retina; the lens and retina are accurately adjusted to each other for ordinary distant vision. For nearer objects, instead of the camera's screw, there is a muscular arrangement for altering the strength or curve of the soft elastic lens, so that it may have more bending power as more divergent rays get in, and so still keep the focus upon the retina. These muscles give us the power of accomodation, or adjustment, for varying distances; and for every slightest alteration in distance we have to make the corresponding but unconscious adjustment. A normal eye, therefore, is one that sees everything beyond fifteen or twenty feet without any muscular effort at all, being naturally adjusted correctly, the picture falling sharply on the retina if only the lids are raised, and the nerve having simply to feel or see the picture. And for all objects within fifteen or twenty feet, the muscular effort of accommodation is involved in rapidly increasing ratio as they approach the eye. Also, every one surely finds some point within which it is impossible to see clearly. This point, called the "near point," represents the full and utmost power of his muscles of accommodation.

It is the abnormal and unconscious overuse and fatigue of this muscular apparatus that underlies the larger part of the weakness and trouble of which people complain. It is not that the eyes tire or are overtaxed in their function of seeing the picture, but it is that, under certain conditions, the muscles are tired and are overtaxed in producing and keeping the pictures for the nerves to see. Three conditions which may produce unnecessary overstrain and fatigue will be worth a moment's explanation. These are old sight, or presbyopia; far sight, or hypermetropia, and uneven sight, or astigmatism. The crystalline lens in youth is quite soft and easily acted upon by the muscles of accommodation, but as early as eighteen years it begins to increase in firmness or hardness, so that the same muscular effort fails to produce the same amount of change in the refractive power of the lens, and the full action of the muscles fails to bring the near point as close to the eye as it used to do. This hardening steadily increases and causes the near point to slowly recede farther and farther off. At sixteen, or so, the near point is usually about two inches from the eye, but as one seldom wishes to hold objects nearer than eight or ten inches, there is a large surplus of reserve power, and the ordinary use of the eyes, on near work, is done with ease. But, as years go by, the near point creeps outward and the eyes are obliged to call upon more and more of their reserve power to accomplish the work they used to do easily. As long as the near point lies well within the working distance, vision can be used almost indefinitely, but when the near point has receded to nine or ten inches, one needs to get almost as near as that to his work, the accommodation is taxed nearly to its utmost, and the strain and fatigue are very great, for no muscle can work at its full power long at a time.

Since the progress of presbyopia, or hardening of the lens and recession of the near point, goes on very gradually, there is no sudden change, no marked symptom to draw one's attention. It will depend greatly, therefore, on the customary length of time the eyes are kept at work at close range, upon the general health, and supply of nervous force not otherwise called upon, whether and when the eyes begin to suffer from old sight. Old sight really begins, therefore, quite early, but does not reach the condition of needing assistance until the near point has gone off to eight or nine inches; it means simply the overtaxing of the muscles of accommodation,—not on account of the work done, but changes in the effort required to do the work; it means improper wear and tear to the nervous system, just in proportion to the demand for near work. Glasses assist this condition by bending the light partially before it reaches the eye, therefore leaving less work for the muscles to do and restoring the near point to a reasonably comfortable position.

Persistence in deferring assistance from glasses is unwise just as soon as conscious fatigue of eyes or head appears. Of course, like all other muscles, proper exercise, when not too long continued, prolongs their power and health, and in that respect it is unwise to put on glasses too early or unless there are distinct and frequent indications of fatigue. The point to remember is, that after reaching the age of forty, decided weariness, lassitude, headache, or pain in the eyes after long-continued close work is probably caused by overstrain of the accommodation, not by difficulties of general health or other reasons, and a moment's testing of the near point will often prove the cause. It follows that when close work,—*i.e.*, from ten to fourteen inches,—involves the use of nearly the full muscular power, the eyes should be frequently rested by looking off to the distance for a few moments; also, if the demands of the day involve several hours of near work, it is of course not best to continue the hard task by reading or writing in the evening, unless aided by glasses. Also, if for any other cause the system is not in good health, then the prolonged and exhausting efforts necessary for the accomplishment of the daily work not only have their injurious effect on the eyes, but use up, or rather waste, the strength needed elsewhere. Although the use of glasses can be deferred for quite a long time after they are really needed, the cost is very real and has to be paid in some way. Just as machinery may be run for some time after oil is needed, and accomplish its work well, it is with a waste of power and damage to the machine.

Again, there is the far-sighted, or hypermetropic, eye, very generally misunderstood. Unlike presbyopia, which develops slowly and becomes evident at middle age, this is a congenital and permanent defect in original shape of the eyeball, which is not deep enough from before backward,—the retina is too close to the lens, intercepting the light before it has reached a focus; a perfectly healthy eye with a lack of proper relative position of its lens and retina. This would naturally cause all its pictures to be blurred and indistinct. But as all eyes have the muscular power for increasing the strength of the lens, such an eye can, must, and does adjust the lens to the faulty position of the retina, and in that way sees perfectly well, but it has to use up more or less of its muscular power to get what the normal eye sees naturally and at rest. The far-sighted eye sees well enough, but not easily. The normal eye is like the rower on smooth water, who utilizes his strength only for progress and rests at will. The hypermetropic or ill-adjusted eye is like one rowing up-stream, who must spend much of his force in overcoming the ceaseless current, and has only the balance for real progress, and to him rest is impossible. The far-sighted eye is, therefore, of necessity, an overworked eye, not on account of what it does, but on account of its shape, for it has first to overcome its ever-present congenital defect, and in addition all the work of the normal eye as well. This constant, unavoidable work shows itself sooner or later in some form of fatigue or nervous exhaustion. There need not be the slightest impairment of vision or pain in the eye, but inability to enjoy continued vision without weariness, fatigue, slight nausea, headache, sick headache, neuralgia, sleepiness, or inability to fix the attention long at a time; or there may be local symptoms of blurring, smarting, aching, or tired feeling in the eyes. Whether or not the hypermetrope experiences any of these symptoms will depend much on his general health, his supply of nervous power, and the amount of close work done. He may escape them all his life, if strong and well; he may be made miserable in health by them, even while in school. Anything that lowers the tone of the system may cause the overtax to declare itself. Very often, however, since the hypermetrope never was able to compare his eyes with others, as to ease of seeing, he remains in entire ignorance that his sight costs him so much, and he may prove a constant patron of the physician for tonics and assistance to combat his various troubles, or else settle down to the belief that he must endure his discomforts as part of his make-up.

To properly care for one's eyes, therefore, one certainly ought to be sure that he was not unconsciously obliging them to bear the burden of a congenital error, and should know the signs pointing to such a defect. Suspicion should be aroused when one finds himself troubled by any of the above-mentioned symptoms or any form of nervous exhaustion, especially if it be noticed to follow continued use of the eyes, either near or far off. Hypermetropia may often be easily detected by testing how far off one can read ordinary print through a lens of known focal strength,—*i.e.*, if one looks through a ten-inch lens and can read clearly at a greater number of inches than the focal length, say twelve, fourteen, or more, then he surely is far-sighted. The lens may be of any strength between six- and eighteen-inch focus, and should be kept close to the eye. The farthest point for distinct vision for a normal eye is at the focal length of the lens.

It is by no means necessary for every one who is hypermetropic to wear glasses, for, if he experiences no signs of nervous trouble or over-fatigue, it is perfectly safe to leave the matter alone, although theoretically he needs help. But the recognition of hypermetropia should put one on his guard, and would supply a positive diagnosis for many forms of nervous difficulty which might arise, and explain many forms of fatigue and disturbance liable to be laid to the brain, the stomach, the liver, etc. Glasses for the hypermetrope simply take off the constant burden at will, do the extra work for him, and give him as fair a chance as normal eyes have. They make him see not a particle better, but easier. They save his accommodation for its proper use, and should be worn enough to make his vision comfortable. Of course, presbyopia will make itself felt much earlier in the far-sighted than in the normal eye.

The third error, very common and but little understood, is astigmatism, another congenital error of shape, this time not of the eyeball, but of the cornea or clear front portion of the eye. The cornea, being a curved surface, acts with the lens as part of the refracting apparatus, and should be like all lenses, truly spherical, but unfortunately it is very often far from true. Most frequently the curve is stronger in the vertical direction than in the horizontal, and these different curves refract the light unevenly, so that the light as it passes through the lens does not all reach the same focus, —*i.e.*, when the light which comes in through the stronger curve is properly focussed, that coming through the weaker curve is not bent so much and is not focussed, so the picture is blurred or streaky. If the eye, by means of its accommodation, adjusts itself for the weaker curve, then the vertical light is not focussed. Such an eye surely cannot rest, for it has to be very active not only for adjusting for different distances, but varying its adjustment constantly, even at the same distance, according as it needs to see the vertical or horizontal parts of the outline; for example, letters being made up of lines in varying directions, the astigmatic eye sees only certain parts of a letter clearly at once, but by rapid changes of accommodation picks them out with difficulty one part at a time. This condition, being congenital, is rarely recognized by the owner except in decided cases, for, having learned as a baby to make the continuous changes in adjustment, and having been obliged to do so all his life, he knows of no other way to see, and supposes he sees naturally; but it costs his nervous system a constant amount of wearying fatigue, and is unavoidable. Using astigmatic eyes may in some senses be compared to riding with oval wheels; one can make progress, but there is much unpleasant jarring and very little ease. Here, again, the results of astigmatism show themselves in some form of fatigue, not in the eye usually, but as pain at the back or top of the head, or in any of the ways already mentioned. There may be hardly any recognized impairment of vision. Astigmatism is not so easily found by the owner of the eyes, but the simplest test is to look at a set of fine radiating lines with each eye separately. If some appear blacker or clearer than others, and change with movements of the head, this error is surely present. Glasses here can be made to even the irregular curves and give the person practically normal eyes to use.

Another very common source of annoyance is the inequality of the eyes: one eye perhaps normal and one somewhat defective. Since the eyes from their mode of enervation must always act equally and together, it follows that one eye does not rest, even if closed or covered, but moves and acts exactly with the other. Also, that if they start unequal by construction, no amount of practice will enable them to adjust themselves separately, but one will always be ill-adjusted and an annoyance, for the brain receives the two impressions, which are unlike, and tries to combine them. Here, again, glasses build the eyes up to equality and ease.

It is often objected that the muscles spoken of are so small, their power so insignificant, their work so automatic, that the annoyance of glasses must far out-balance the benefit, and that certainly a man in ordinary health should not miss the trifling nervous power needed to overcome such errors. But it is to be remembered that the hair-spring of a watch is quite as important as the main-spring, and its ill-adjustment affects the whole watch. It is not the amount of the work done, but the continued obligatory action that is the starting-point of the disturbance. Every muscle needs some rest, but in these optical errors there is no rest unless the eyes are closed or good vision abandoned. Rest to the hypermetropic or astigmatic eye means loss of focus and blurred sight, which will not be tolerated as long as one's will-power can hold out. Rest to the normal eye means rest, and yet perfect vision everywhere beyond fifteen or twenty feet. A blacksmith hammers all day with his heavy hammer because he rests between the blows, but to hold a feather at arm's length for half an hour is next to impossible, and the attempt to do so would disturb him in many more ways than the tired arm, and involve the whole power of his will and attention to prevent the longed-for relaxation.

But fatigue in its many forms is not the only result of these optical errors, for an increase in muscular work anywhere means an increased call for blood, and this, kept up abnormally strong and long, tends to induce in many cases chronic congestion. In the eye this may show itself either in the lids or their appendages, or may result in deeper-seated, less visible, but more serious physical conditions. When this congestion affects the lids, it appears by smarting, pricking, gravelly or burning feelings after use of the eyes, or in sensations of fulness, heaviness, sleepiness, or nervous twitching of the lids. It may affect the edges chiefly and make them red and scaly, with more or less loss of lashes. All these symptoms in lids may also result from truly local forms of irritation, as will be mentioned later, but more often some optical error lurks at the bottom, and this should be strongly suspected when the condition recurs after relief has once been obtained by local treatment.

The position of the head often necessary in the work of the dentist may sometimes induce, and at least would greatly aggravate, the tendency to congestion. Unless he uses the mirror largely, a few moments of erect position, thrown in frequently, would do much to avoid trouble.

The common complaint and anxiety in regard to floating black specks or spots is usually due to slight and temporary congestion of the back of the eye. In themselves these spots mean little or nothing, unless they are very abundant, large, or persistent. They are but the shadow of little particles which are present in the fluids of almost every eye, but the healthy eye does not notice them. If, however, from indigestion or any other cause, or more frequently from the ever-present strain of the overtaxed accommodation, congestion of the deeper parts of the eye is induced, then the retina becomes sensitive and irritable and notices them. Just as the sore finger seems to come in contact with everything, yet it is only because it is more sensitive that it demands attention to the touches usually unheeded.

Photophobia, or dread of light, is another symptom of the same sort. Very often it is only a sign of irritation from some optical cause. Though this dread of light is present in nearly all inflammatory diseases of either the front or back parts of the eye, it is usually then accompanied by other more serious symptoms, as pain, tenderness on pressure, glimmering, flashes of light, dark clouds in permanent positions, impairment of sight, or some evident external signs of trouble needing medical advice. Just as the tired, overtaxed person becomes nervous and irritable and is fretted by trifles not ordinarily noticed, so the eyes, tired and doomed to constant unnatural work, become sensitive and annoyed by many things unnoticed by the properly adjusted eye. When the eyes are normal in action and have no local diseases, it is seldom that they object to severe and long-continued work, even if the general health is far from good, for they do get some rest. It is the weak links with flaws in them that give out under strain, and if the eyes begin to grumble there must surely be a reason. It is well, therefore, for every one who uses the eyes much to heed the early signs of fatigue or nervous irritation felt anywhere and see first whether, as is very likely, there is not some abnormal friction in the working of the eyes, which can be so easily and surely found, proved, and corrected, rather than search among uncertain possibilities elsewhere, or deny himself the free use of so valuable and willing servants. For if we review for a moment the long list of results following optical errors, smarting, burning, gravelly sensations, reddened and irritable eyelids, weak, tired feelings, headache, sick headache, pain in the eyes, blurring, twitching, sleepiness, itching, etc., etc., we find strangely that they include nearly all the complaints that are usually made, and experience proves that these errors, though not the only possible causes, are the most frequent and likely.

The question why there are so many defective eyes found nowadays deserves a moment's consideration, and has two answers: First, we know that in the organic world nature always works towards a perfect pattern in shape as well as function, but seldom attains it, and the result is infinite variety with but slight variations. Now, in all other instances, exactness of shape or form is not a necessary requisite for health or perfect function, but in the eye, as an optical instrument, it is the perfection of the curves and the accuracy of position of lens, cornea, and retina that give the perfect function of producing accurate pictures for us to see. But variations from the perfect standard are nearly as common in the eye as in the features or elsewhere in the body, and here interfere directly with the value of the organ; the burden of overcoming the fault falls upon the muscles of accommodation, which do their very best till compelled by exhaustion to complain. There is therefore nothing strange in the prevalence of such errors. Secondly, experience is unexpectedly showing the results of this overstrain and tax to be so varied and far-reaching that errors are looked for now where a few years ago they would not have been suspected, and the key to many a puzzle is found. This must be my apology for making this the prominent part of this paper; and yet there is one other reason, namely, the fact that both hypermetropic and astigmatic errors can be detected, measured, and proved by strictly objective or mechanical means, and thus there is no chance for doubt as to diagnosis. Errors cannot be found where they do not exist, though it is of course possible to overlook slight ones, and if errors are found the results are inevitable. Yet the mistaken idea which is so prevalent is that there must be some impairment of vision if error is present. To see well is one thing, but to see well and easily is quite another.

Myopia, or near-sight, has been purposely omitted, for, being in some sense a disease which affects the eye in childhood and youth, reaching a stand-still usually at the age of twenty-two or twenty-three, the time for attention to that form of optical error is while it is in progress and more or less amenable to care and treatment, and therefore is not of importance for you now. After myopia reaches its fixed condition, though a misfortune in many ways, it is not wholly an unfavorable one for the dentist, and does not lead often to trouble, unless complicated with other errors or of high degree. Myopia really is the giving way or bulging backward of the abnormally weak back part of the eye,—the eyeball becomes too long or egg-shaped, thus carrying the retina beyond the proper focus of the lens, and, there being no power of weakening the refraction, distant vision is permanently impaired. Contrary to the popular belief and the comfortable hopes of many, vision does not improve with age. Presbyopia comes on as usual, but is modified by the condition present, so that glasses for reading are not needed as early as usual, or in some cases never needed. This gain, however, is offset by the life-long loss of distant vision.

There are, of course, very many cases where the conjunctiva lining the lids or their edges may show simply local forms of congestion, interfering greatly with their comfort, and brought on by various causes, such as exposure to excessive light, dust, wind, smoke, close air in ill-ventilated rooms or crowded halls, or, indirectly, by long-continued work with insufficient light, lack of exercise, etc., but such cases are apt to be temporary and vary with the action of the cause. All these symptoms are aggravated by close work or by artificial light. There is quite possibly even here some slight optical error that only prepares the way for the influence of these as secondary causes. Where none is found, the simplest remedies for the smarting, itching, fatigue, etc., are the use of mild astringent washes, such as the ordinary borax wash, ten grains to the ounce of water or camphor-water, or in more pronounced cases from one-half to two grains of sulphate of zinc instead of the borax; these applied three or four times a day. Also, the application of heat or cold, as may be most agreeable. These latter remedies, however, need to be used properly, for they are more powerful sometimes than is supposed. The object in these cases is, of course, to reduce congestion, and although this may be accomplished either with hot or cold water, the exact opposite can also be done with either, so that simply to advise heat or cold might result in anything but relief. For example, if the hand be put into very cold water a short time only and then removed, the reaction is so strong that very soon the hand becomes red and hot or congested; if, however, it is put into cool water a little below the temperature of the blood, and left a little longer, there is no reaction, but the temperature is lowered and the blood driven away for quite a little while. So also with heat; warmth draws blood to a part, but stinging heat acts powerfully in reducing congestion, and, as is well known, is a very valuable aid in checking hemorrhage. Therefore, stinging hot water should be used to bathe the eyes, or, if preferred, one or two thicknesses only of cloth wet in cool water laid over the eyes and kept wet, so as to gradually abstract heat by evaporation. Warm water only increases the flow of blood, and a pad of cloth wet in cold water quickly turns into a warm poultice. Position, also, has much to do with the result; if the head is bent down over the bowl and the water splashed upon the eye, both the position and the shock of the splashing tend to produce congestion, and the effect of the application is annulled. The hot water should be applied on a large cloth or sponge, with the head erect or reclining, and gently laid in the eyes and kept quietly there as long as the stinging heat remains, then renewed. This is a very refreshing application to tired eyes and head, but of course should not be used too frequently. Often a congested or swollen state of the conjunctiva, by extending into the fine tear-ducts, causes a very annoying overflow of tears, especially in bright light, wind, or when the head is bent forward. Patient use of mild astringents and occasional application of heat, with the removal of any discoverable cause for the congestion, optical or otherwise, will usually bring about relief, but in many cases there is a real contraction or obstruction of the ducts, which, of course, calls for local treatment.

In regard to artificial light of various kinds, it is necessary simply to remember its prominent differences from daylight. Daylight is diffused light, proceeding, of course, primarily from the sun, but every object and the whole sky become also sources of illumination, so that the light is more even and shadows much softer. Direct sunlight we seldom permit to enter the eye. Artificial light is vastly less intense, so that the retinal pictures are much fainter and tax the seeing power of the eye more; and also, for the same reason, the pupil of the eye has to expand greatly to let in more light. This large pupil, from optical necessities, makes the focussing of the light less accurate and clear, just as in a camera the small stop with brilliant light will give the best defined pictures. Again, the light usually comes from one or more brilliant sources not far off, and direct light from these is apt to enter the eyes, or perhaps only one. This annoys and worries the nerves controlling the size of the pupil, for there is a distracting desire to dilate for the dimmer parts of the picture and to contract on account of the strong light from the flame. It is not the quantity of light which is apt to fret the eye, but the inequality in the illumination. Strong contrasts are fatiguing: a streak of sunlight on a light wall is more fatiguing than full sunlight over the whole surface, for the eye can easier accommodate itself. From this it follows that the sources of artificial light should not be visible while at work,—no direct light should get into either eye. With this point attended to, the more light the better. The preferable position for the light is above and a little behind the head. As to the kind of light, the important points are brilliancy and steadiness. Arc lights fail greatly in steadiness and so are not good for close work, and their excessive brilliancy makes their direct rays very trying. Incandescent lights, if shielded, are as desirable as any known, for they have brilliancy and steadiness and the additional advantages of lack of heat and contamination of the air. One other peculiarity of artificial light is the predominance of the yellow rays, which are more irritating to the retina than the mixed white light. For this reason sensitive eyes often find great relief in using a chimney or shade of a very light blue tint; this neutralizes part of the yellow rays and gives a whiter and more agreeable light, like daylight.

The influence of tobacco perhaps deserves a moment's notice, though, probably on account of moderate use, but few in the dental profession run much risk from this cause. Still the danger from it is often so insidious and depends so little on what is considered an excessive use, that it is well to understand the matter clearly. Tobacco, as is well known, not only has its pleasant qualities which win so many adherents to its use, but it has very active poisonous qualities, which the system only learns to tolerate, just as it does opium, arsenic, and many other deleterious substances. It depends entirely upon the variable ability to absorb and eliminate the poisonous elements. If one absorbs a certain quantity daily, and is able to eliminate the same, the effect of tobacco will not be likely to be harmful; but if from any cause, or at any time, the power of elimination falls below the amount absorbed, then the system begins to be loaded or saturated with the drug, and, like a tank with ill-adjusted inflow and outflow, sooner or later it will suddenly overflow,—that is, unexpectedly the system will begin to suffer somewhere from the accumulated absorption of a long time. In the case of tobacco it may be the heart, the digestion, or the nervous system that will suffer first, or it may be the optic nerve will feel the full force and begin to wither and atrophy. If so, vision begins to fail in both eyes, everything acquiring a smoky, hazy look. This steadily increases without pain or annoyance, and unless one attends to it and the process is checked before it gets much headway, permanent loss of vision is quite sure to result. The danger is in its gradual, stealthy progress, without pain or symptom calling attention until vision is much impaired, and this, in those who have long been users of tobacco, and perhaps in only moderate degrees. The evils of the use of tobacco come, therefore, not in those who are beginning its use, not necessarily in those using it to excess, but in any one, at any time, whenever from one cause or another elimination is impaired. It is especially apt to show itself when the condition of the system is lowered by loss of sleep, anxiety, trouble, or decided change in habits, or by the habitual use of stimulants. The treatment is entire cessation from tobacco in every form, so that the whole power of the system may be used for elimination. One can usually retain what vision he has left when he commences treatment, but not surely.

On the whole, the eyes are much like other parts of the body,—when they are in order, we use them, hardly knowing that we have them, thinking little how they do their work, unaware of the many intricate processes and marvellous accuracy involved. Looking out over a broad landscape, or any comprehensive scene, one seldom realizes that the whole picture in the eye is but about an inch square; or as one looks across a broad street and recognizes an acquaintance, noticing all the minutiæ of his expression and dress, down even to the cord of an eye-glass, it is difficult to comprehend that the real picture seen is only as large as the letter "i" in small print. Any one who has used a microscope will understand what nice adjustment is necessary for such minute pictures, and, as we are constantly looking at objects at varying distances, and have to adjust rapidly, yet perfectly, for each slightest change, and, moreover, have two eyes and two pictures which must fall on exactly corresponding portions of the retinæ, or else give rise to very distressing double vision, also having to follow moving objects receding or advancing,—with all these complications, it is not hard to perceive that the work of the muscles of the eyes is not only very nice but very lively work. It is really remarkable how well and how untiringly fairly normal eyes do all this without fatigue or complaint. But if in addition they are burdened with additional obligatory and continuous work on account of congenital errors of shape, even then they do their best, but sooner or later the system feels the strain and has to pay the cost of the overtax.

Since normal eyes do so well, and so rarely complain, surely it is worth while to take some thought as to their proper care, heed the early signs of fatigue as one would the squeaking of a machine, and relieve it, if possible, not by stopping its use, but by removing the annoying friction. It is by no means claimed that the overtaxing of the muscles of accommodation is the sole cause of all the symptoms of nervous fatigue, but it is surprisingly often the exciting cause. The lowered tone of the general health, and the prevalent over-sensitive condition of the nervous system, are generally prominent elements; but granting this, it must still remain the surer plan, if even adopted as a temporary remedy, to reduce by positive means the unnatural strain inevitably caused by these optical errors, rather than seek by uncertain methods to build up the health to the ability to bear the unnecessary burden.

